# Tuboovarian Abscess due to Colonic Diverticulitis in a Virgin Patient with Morbid Obesity: A Case Report

**DOI:** 10.1155/2012/413185

**Published:** 2012-08-16

**Authors:** Zafer Selçuk Tuncer, Gokhan Boyraz, Senem Özge Yücel, İlker Selçuk, Aslıhan Yazicioğlu

**Affiliations:** ^1^Gynecologic Oncology Unit, Department of Obstetrics and Gynecology, Hacettepe University Faculty of Medicine, 06100 Ankara, Turkey; ^2^Department of Obstetrics and Gynecology, Hacettepe University Faculty of Medicine, 06100 Ankara, Turkey

## Abstract

Since tuboovarian abscess is almost always a complication of pelvic inflammatory disease, it is rarely observed in virgins. A 30-year-old virgin patient presented with pelvic pain, fever, and vaginal spotting for the previous three weeks. Her abdominopelvic computed tomography scan revealed bilateral multiseptated cystic masses with prominent air-fluid levels suggesting tuboovarian abscesses. The sigmoid colon was lying between two tuboovarian masses, and its borders could not be distinguished from the ovaries. The patient was presumed to have bilateral tuboovarian abscesses which developed as a complication of the sigmoid diverticulitis. She was administered intravenous antibiotic therapy followed by percutaneous drainage under ultrasonographic guidance. She was discharged on the twenty second day with prominent clinical and radiological improvement. Diverticulitis may be a reason for development of tuboovarian abscess in a virgin patient. Early recognition of the condition with percutaneous drainage in addition to antibiotic therapy helps to have an uncomplicated recovery.

## 1. Introduction

A tuboovarian abscess reflects an agglutination of pelvic organs including the tube, ovary, and bowel forming a palpable complex. It represents an end-stage process of acute pelvic inflammatory disease. Tuboovarian abscess has been reported to complicate 18–34% of patients with pelvic inflammatory disease [1, 2]. Risk factors for tuboovarian abscess are similar to that of pelvic inflammatory disease and include multiple sexual partners, intrauterine device, and low socioeconomic status [3]. Since the disease commonly is caused by the sexually transmitted microorganisms, intercourse with a partner having infection is the most important risk factor in tuboovarian abscess formation. However, gynecologic surgery, genital malignancy, in vitro fertilization treatment, and perforated appendicitis have also been shown to cause tuboovarian abscess in the literature [4–7].

Diverticulosis is a common condition in elderly population. It is defined as a pocket of mucosa which herniates through areas of weakness usually at vascular entry sites [8]. One of the complications of diverticulosis is diverticulitis which will develop in approximately 10% of the patients [9]. Occasionally acute diverticulitis will involve the female reproductive organs which lie in close proximity to the sigmoid colon resulting in tuboovarian abscess. 

A case of tuboovarian abscess associated with diverticulitis and treated with percutaneous drainage in addition to antibiotic therapy in a young virgin patient with morbid obesity was reported.

## 2. Case Presentation

A 30-year-old virgin patient presented with pelvic pain, fever, and vaginal spotting for the previous three weeks. She was also suffering from malodorous vaginal discharge and constipation. Her past medical history was unremarkable except for morbid obesity. The patient was 160 kg with a BMI of 35.5.

Physical examination demonstrated fever (39°C) and mild tachycardia (110/minute) with bilateral lower abdominal quadrant tenderness. Her blood analysis revealed an elevated white blood count (20.600/mm^3^) and anemia (8.5 g/dL). Her erythrocyte sedimentation rate was 90 mm/h. Pelvic ultrasonography showed bilateral cystic pelvic mass, but the result was considered to be unsatisfactory due to morbid obesity. CA 125 level was 233 IU/mL. Her blood and urine cultures were found to be negative. 

**Figure 1:**
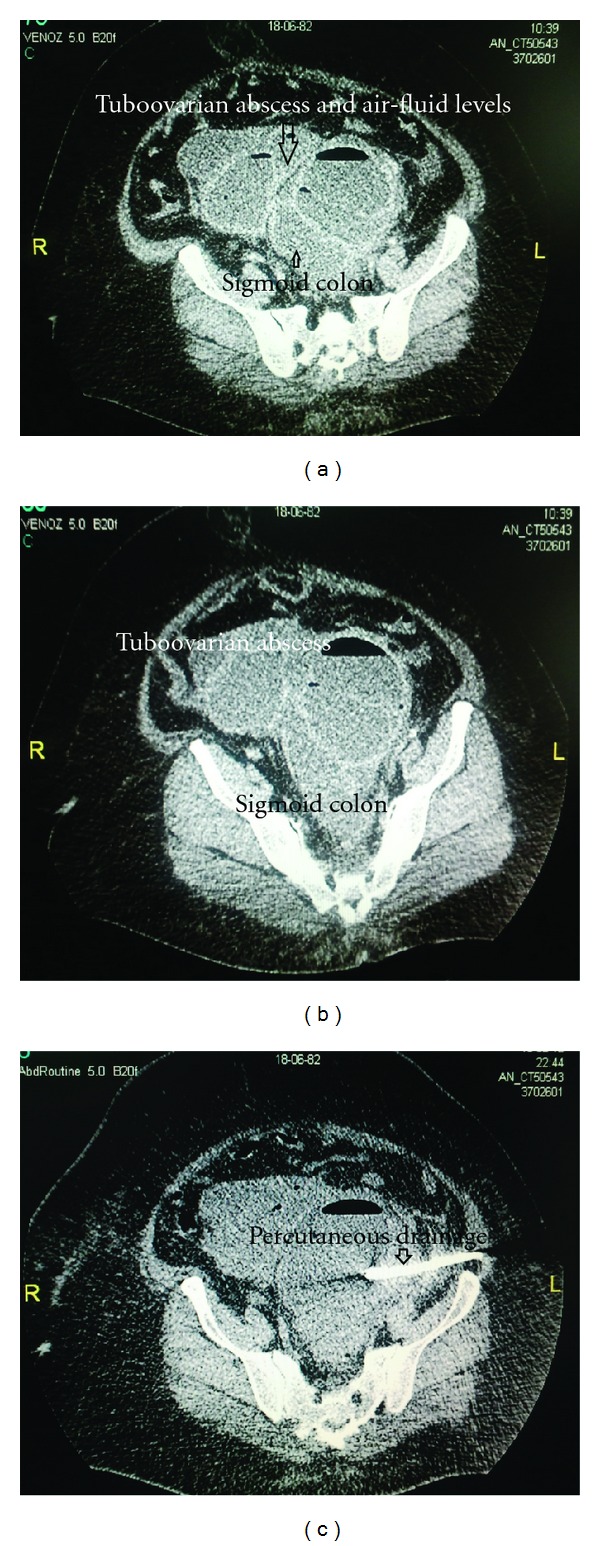
(a) The sigmoid colon was lying between two tuboovarian masses, and its borders could not be distinguished from the ovaries. (b) The sigmoid colon was lying between two tuboovarian masses. (c) Percutaneous drainage.

Her abdominopelvic computed tomography scan revealed bilateral complex adnexal masses. The left and right lesions were measured to be 19 × 11 cm and 8.5 × 10 cm in size, respectively. Both of the lesions were multiseptated with prominent air-fluid levels suggesting tubo-ovarian abscesses (Figures 1(a), 1(b), and 1(c)). The sigmoid colon was lying between two tuboovarian masses, and its borders could not be distinguished from the ovaries. The mesentery of the sigmoid colon was also found to be thickened due to inflammation. The uterus was found to be normal without any sign of pelvic inflammatory disease. 

The patient was presumed to have bilateral tuboovarian abscesses which developed as a complication of the sigmoid diverticulitis. Intravenous fluid replacement was started, and oral intake was avoided. Since the patient had fever, tachycardia, and elevated white blood count, she was administered piperacillin-tazobactam by the department of infection diseases considering the risk of sepsis with a dose of 4.5 g four times a day intravenously. Percutaneous drainage under ultrasonographic guidance was performed using two drainage catheters. A total of 1000 mL purulent material was aspirated from both lesions. The catheters were left in place for further drainage. Three different bacteria including *Escherichia coli*, *Veillonella,* and *Peptostreptococcus* grew on the cultures of the abscess drainage fluid. During the followup, the clinical condition of the patient gradually improved. She became afebrile, and her white blood count returned to normal level. She was allowed oral intake on the 7th day of hospitalization. Serial ultrasonograms were performed, and decrease in abscess sizes was noted. On the 19th day of hospitalization, the drainage catheters were removed. The patient was discharged on 22nd day of hospitalization with prominent clinical and radiological improvement.

## 3. Discussion

Since tuboovarian abscess is almost always a complication of pelvic inflammatory disease, it is rarely observed in virgins. However, a review of the literature revealed that tuboovarian abscess may also develop due to other rare causes including poor perineal hygiene, previous gynecologic surgery, coexisting genital malignancy, appendicitis, and diverticulitis [4–7].

The possibility of diverticulitis as an etiology of tuboovarian abscess is not usually considered initially. Diverticulitis is usually observed in elderly population, but it may also be diagnosed in younger patients with less incidences. According to [10], the 55–66% of the women with diverticular disease were over 80 years of age and only 10% were younger than 40 years of age. Recently, the frequency of diverticular colonic disease increased in patients younger than 50 years of age. 

A 31-year-old woman was reported to undergo left salpingo-oophrectomy for a tuboovarian abscess, but she underwent a second surgery because of a recurrent pelvic abscess due to underlying diverticulitis which was undiagnosed at the first operation [11]. Diverticulitis with abscess formation can be very insidious, and reports of presentation as only chronic diarrhea and even brain abscess have been described [12]. A recent case of 63-year-old patient with diverticulitis misdiagnosed to have a primary gynecological abscess subsequently developed colonic perforation [13].

Pelvic pain, signs of infection, and mass at ultrasonography in the presented case easily made the diagnosis of bilateral tuboovarian abscess. Since the patient is virgin, rare causes of tuboovarian abscess were investigated. Computed tomography scan revealed the diagnosis of diverticulitis complicated by tuboovarian abscess [14]. Conservative management with antibiotic therapy and percutaneous drainage resulted in significant clinical improvement of the patient. Since the pelvic inflammatory infection is usually caused by the sexually transmitted microorganism *N. gonorrhoeae* and *C. trachomatis*, isolated microorganisms further confirmed the diagnosis of abscess resulting from diverticulitis. 

## 4. Conclusions

Diverticulitis may be a reason for development of tuboovarian abscess in a virgin patient. Early recognition of the condition with percutaneous drainage in addition to antibiotic therapy helps to have an uncomplicated recovery.
